# Machine learning classification of resting state functional connectivity predicts smoking status

**DOI:** 10.3389/fnhum.2014.00425

**Published:** 2014-06-16

**Authors:** Vani Pariyadath, Elliot A. Stein, Thomas J. Ross

**Affiliations:** Neuroimaging Research Branch, Intramural Research Program, National Institute on Drug Abuse, National Institutes of HealthBaltimore, MD, USA

**Keywords:** biomarkers, fMRI, machine learning, nicotine addiction, support vector machines

## Abstract

Machine learning-based approaches are now able to examine functional magnetic resonance imaging data in a multivariate manner and extract features predictive of group membership. We applied support vector machine (SVM)-based classification to resting state functional connectivity (rsFC) data from nicotine-dependent smokers and healthy controls to identify brain-based features predictive of nicotine dependence. By employing a network-centered approach, we observed that within-network functional connectivity measures offered maximal information for predicting smoking status, as opposed to between-network connectivity, or the representativeness of each individual node with respect to its parent network. Further, our analysis suggests that connectivity measures within the executive control and frontoparietal networks are particularly informative in predicting smoking status. Our findings suggest that machine learning-based approaches to classifying rsFC data offer a valuable alternative technique to understanding large-scale differences in addiction-related neurobiology.

## Introduction

Conventional univariate methods of fMRI analysis have been used to identify differences in neural processing between various diseased populations and healthy controls over a plethora of tasks. However, not all such group differences are guaranteed to be *predictive*; there may be significant overlap between the two group distributions of the pertinent metric. Further, traditional univariate approaches to fMRI analysis by definition overlook multivariate patterns in the data. Machine learning offers a variety of tools to address the above limitations. Support vector machine (SVM)-based algorithms (Vapnik, 2000), for example, have been used successfully to identify neural patterns of activation (Haxby, 2012) as well as for group-level differences (Craddock et al., 2009).

Attempts to apply machine learning-based approaches to classify individuals based on various disease states has gained significant traction for screening and diagnosis (Vemuri et al., 2008; Stonnington et al., 2010), and monitoring disease trajectory (Hobbs et al., 2010). Applications of machine learning to this end include classification of schizophrenia from task activation maps (Demirci et al., 2008), using structural images to classify individuals as addicted or not (Zhang et al., 2005), and using task activation maps to classify schizophrenia, Alzheimer's, and mild traumatic brain injury (Ford et al., 2003). Depending on the specific method involved, and the neurobiological disease in question, such attempts have been met with moderate to high success, i.e., ranging from 60 to 100% classification accuracy (Orrù et al., 2012).

Addiction in particular stands to benefit from the application of machine learning-based approaches. Multiple gene × environment interactions go into determining susceptibility to addiction at various stages—initiation of drug use, transition to repeated drug use and then on to compulsive use (Kreek et al., 2005). Aside from this, the drug of abuse itself interacts with neural systems to modulate drug-related circuitry and resulting cognition (Volkow et al., 2009). Not surprisingly, multiple brain networks have been implicated in this complex disease, and, to date, very few neural biomarkers have been identified for predicting vulnerability to addiction and treatment outcome (Pariyadath et al., 2013). Importantly, with rare exceptions (Zhang et al., 2005), the search for such biomarkers through neuroimaging has thus far been restricted to univariate approaches. Through a multivariate approach, we can begin to identify complex interactions within and between brain networks, and some day explore parallel contributions from genetic and environmental sources to neural function.

When comparing groups, differences in task performance can sometimes confound the interpretation of differences in neural activation patterns. Of late, resting state functional connectivity (rsFC) analysis has proven immensely valuable for extracting differences in neural function in the absence of an explicit task. Functional connectivity analyses indicate that there are significant differences in neural architecture and functioning in substance dependent individuals (Sutherland et al., 2012). This analysis approach has been combined with machine learning tools to extract a neural metric for maturity (Dosenbach et al., 2010) within a healthy cohort. Of relevance here, SVM-based approaches have been shown to be successful in classifying major depressive disorder (Craddock et al., 2009) and schizophrenia (Shen et al., 2010) from rsFC data. To date, however, rsFC data has not been explored using machine learning in addicted individuals.

In this study, we sought to identify neural features that may explain addiction in a multivariate fashion—those that speak to addiction when examined in tandem as opposed to in isolation—and that are *predictive* of the addicted state. To this end, we applied a linear SVM-based method to rsFC data from nicotine-dependent individuals and controls. Linear SVM algorithms have been shown to be an effective approach for large-dimensional problems, especially those where the number of features exceeds the number of samples (Hsu et al., 2003). We employed a network-centered approach, capitalizing on a previous attempt at reducing neural activity to resting state networks (Smith et al., 2009). Recent research suggests that cognition, and psychopathologies thereof, may be better understood as involving distributed brain areas that function as part of large-scale networks, as opposed to a single, focal brain region (Bressler and Menon, 2010). Other complex diseases, such as Alzheimer's, major depression, schizophrenia, and autism have benefited from parcellating the brain in terms of large-scale functional networks (Bressler and Menon, 2010). Further, this approach permitted us to explore differences in functional connectivity without constraint only to regions that have previously been identified through univariate approaches to be relevant to addiction. We compare three different network-centered measures to assess the—(1) the extent to which each node within a network is representative of the parent network, (2) functional connectivity between different nodes within a network, and (3) functional connectivity between different networks.

## Materials and methods

### Participants

Participants included 21 smokers (9 female) and 21 non-smoking controls (11 female), whose details are shown in Table 1. All smokers scored at least 6 on the Fagerstrom Test for Nicotine Dependence (FTND). Individuals with a history of pre-morbid neurological disease, major medical, or axis I psychiatric diagnosis other than substance use disorder, assessed by the computer-administered Structured Clinical Interview (SCID) for the Diagnostic and Statistical Manual of Mental Disorders IV (DSM-IV) screening version and clinician interview, or who had current substance dependence other than nicotine or cannabis, based on DSM IV criteria, were excluded from the study. Smokers were allowed to smoke *ad libitum* prior to the scan session. Participants gave written informed consent to this study approved by the Institutional Review Board at the National Institute on Drug Abuse-Intramural Research Program.

**Table 1:** **Demographic characteristics of study population**.

	***Smokers***	**Controls**	***P*-value**
Number	21	21	
Age (mean ± *SD*)	38.19 ± 9.79	39.90 ± 10.82	0.60^a^
Gender			
Male	12	10	0.38^b^
Female	9	11	
Race/Ethnicity			
White	11	7	0.33^b^
Black	8	12	
Hispanic	1	1	
Unknown	1	1	
FTND	6.86 ± 1.04	–	

a P-values were obtained by a two-sample two-tailed t-test.

b P-values were obtained by a two-tailed chi-squared test.

### fMRI acquisition and pre-processing

During resting state scans, participants were instructed to rest and keep their eyes open but not to think about anything in particular. Functional MRI data were collected on a 3-T Siemens Allegra MR scanner (Siemens, Erlangen, Germany) equipped with a quadrature volume head coil. Thirty-nine slices were acquired positioned at 30° to the AC-PC line and were prescribed to cover the whole brain. The data were acquired using a single-shot gradient-echo echo-planar imaging (EPI) sequence with repetition time (TR) of 2 s, echo time (TE) of 27 ms, flip angle (FA) of 78°, field of view (FOV) of 220 × 220 mm, and an in-plane resolution of 3.44 × 3.44 mm with thickness 3.5 mm. For registration purpose, high-resolution anatomical images were acquired using a 3D magnetization prepared rapid gradient-echo (MPRAGE) T1-weighted sequence with TR of 2.5 s, TE of 4.38 ms, FA of 7°, and a voxel size of 1 × 1 × 1 mm.

Data processing and analyses were conducted in AFNI (Cox, 1996). Preprocessing included slice-timing and motion correction. Data were inspected for motion using censor.py (http://brainimaging.waisman.wisc.edu/~perlman/code/censor.py), employing a censoring threshold of 0.3 mm for translation and 0.3° for rotation between consecutive TRs. Data were then spatially normalized to the standard Talairach space. Spatial smoothing to a 6 mm FWHM Gaussian kernel was performed to increase spatial signal to noise ratio. Global fluctuations, originating presumably from such systemic effects as respiration and cardiac-induced pulsations, were accounted for individually by orthogonalizing the time-courses with respect to the first three principal components from the white matter voxel time course ensemble and the first three principal components from the time course ensemble of the cerebrospinal fluid (CSF) voxels (Behzadi et al., 2007). In addition to these physiological regressors, participants' time courses were also orthogonalized with respect to the six motion parameters. Time courses were band-pass filtered (0.01–0.15 Hz) to retain only the low frequency components in the signal. Although it is common to use a more narrow frequency band (e.g., cutoff frequency = 0.08 Hz), many studies do employ a higher cutoff frequency, such as 0.15 Hz, and in some rsFC analysis methods, a broader frequency band might even be preferable (Wu et al., 2008; Braun et al., 2012). We therefore chose to employ a broad frequency range for our network-based analysis. To address any concerns that findings here may be driven by physiological noise (stemming from the use of this frequency range), we tested the classifier after band-pass filtering the signal with a lower cutoff frequency (0.1 Hz). We did not observe any significant difference in classifier performance.

Recently, there has been some concern regarding motion-related artifacts in rsFC computation, specifically manifesting as decreases in estimated long-distance connectivity as a result of increased head motion (Power et al., 2012). To ensure that our results were not artifacts induced by head motion, we were careful to remove volumes with head motion above a stringent threshold during the pre-processing stage (0.3 mm for translation and 0.3° for rotation between consecutive TRs), and time courses were orthogonalized with respect to the participant's motion parameters. Additionally, we computed the root mean squared (RMS) head position change or for smokers and controls, and also the final number of volumes that were included in the computation of correlation coefficients.

### RSN node selection

**Figure 1:**
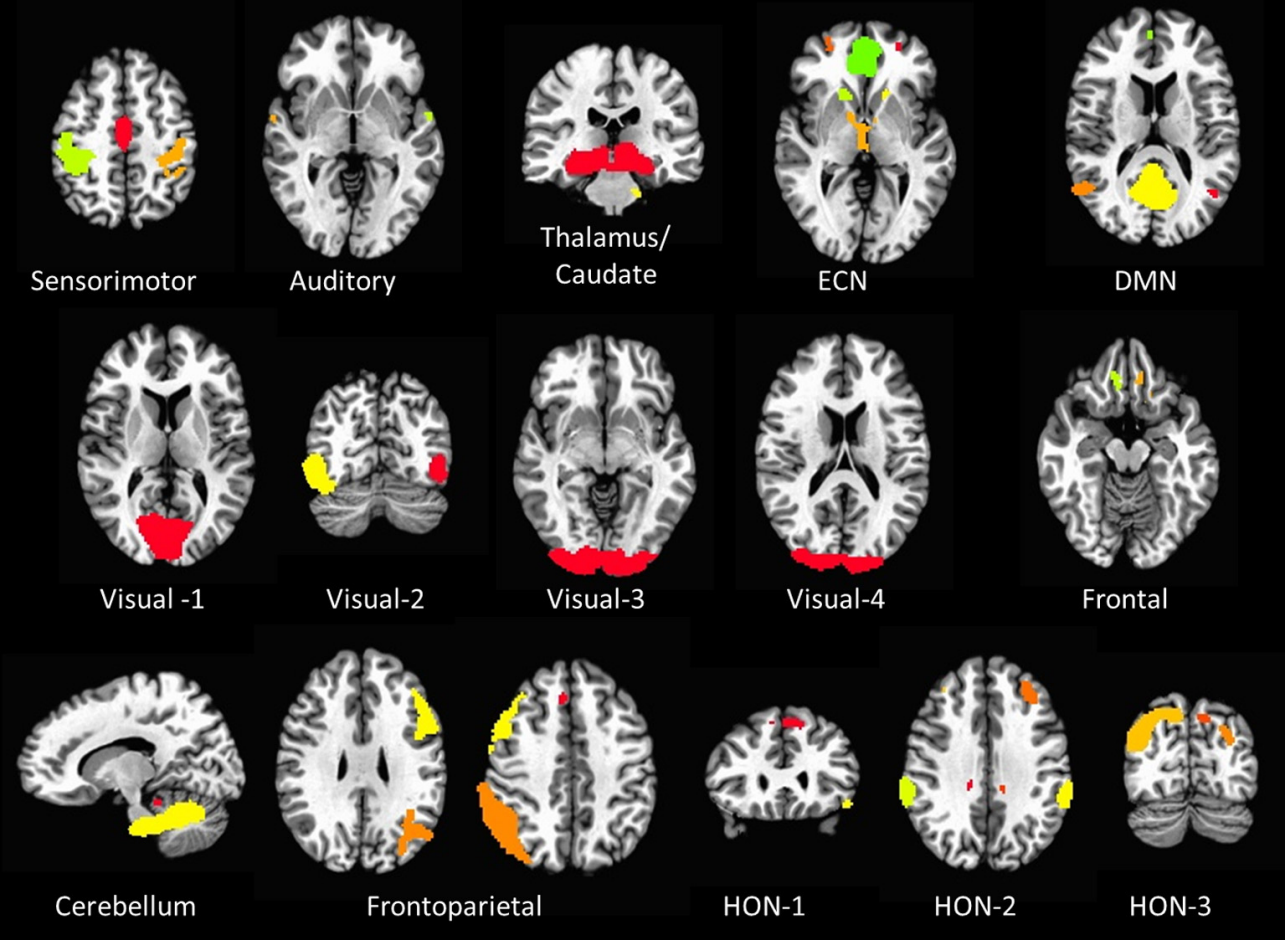
**The 16 resting state networks and their corresponding node regions**. Resting state networks were selected and thresholded from a 20-component ICA decomposition of task fMRI data from the BrainMap database and resting data from 36 participants carried out in a previous study (Smith et al., 2009) (DMN, Default Mode Network; ECN, Executive Control Network; HON, Higher Order Network).

Sixteen resting state networks (RSNs) were selected from a 20-component ICA decomposition of task fMRI data from the BrainMap database and resting data from 36 participants carried out in a previous study (Smith et al., 2009). Four RSNs were discarded from the original 20 as they had previously been identified as artifactual. Of the 16 spatial maps, four were not categorized in the original study. Three of them were speculated to overlap with multiple other RSNs—specifically sensorimotor, frontoparietal, and executive control networks (ECNs); we refer to these three here as Higher Order Networks or HONs (Figure 1). The fourth one comprises the cuneus and surrounding occipital regions, and is categorized here as Visual-4 (Figure 1). The 16 spatial maps were reduced to 56 node regions by thresholding at *Z* = 6 with a minimum cluster size restriction of 50 (1 × 1 × 1 mm^3^) voxels (Figure 1; Table 2) using the AFNI program 3dROIMaker (Taylor and Saad, 2013). This level of thresholding was chosen so as to qualitatively capture the networks observed in Smith et al. (2009), as these networks consistently appear in the literature and are temporally stable (Damoiseaux et al., 2006; Chen et al., 2008).

**Table 2:** **The 16 RSNs and their corresponding node regions**.

**Node #**	**RSN**	***X***	***Y***	***Z***	**Volume (# of voxels)**
**SENSORIMOTOR**					
1	Right postcentral gyrus	41	−27	44.0	7312
2	Bilateral paracentral gyrus	1.5	−13.9	43.5	4016
3	Left postcentral gyrus	−38.6	−28.2	44.3	3752
**AUDITORY**					
4	Left superior temporal gyrus	−56.3	−2.3	−0.3	144
5	Right superior temporal gyrus	59.7	−3.4	−0.2	120
6	Left superior temporal gyrus	−60.8	−23.2	10.6	72
**THALAMUS/CAUDATE**					
7	Thalamus/Caudate	3.4	−24.9	−8.4	29,552
8	Left culmen	−15.7	−26.3	−29.3	208
**EXECUTIVE CONTROL NETWORK**					
9	Right anterior cingulate	1.7	33.3	13.3	18920
10	Left superior frontal gyrus	−26.4	44	19.2	5960
11	Right middle frontal gyrus	30.1	45.6	18.6	4696
12	Right caudate	15.4	16.3	0.8	728
13	Left caudate	−14.6	16	0.9	384
14	Bilateral thalamus	3.9	−11.4	4.3	1888
**DEFAULT MODE NETWORK**					
15	Bilateral posterior cingulate	1.2	−57	21.6	25376
16	Left middle temporal gyrus	−44.2	−64.7	24.6	3432
17	Right superior temporal gyrus	51.4	−59.1	19.7	2472
18	Bilateral anterior cingulate	2	50.7	−0.9	1984
**VISUAL-1**					
19	Bilateral lingual gyrus	2.1	−74.5	4.1	45072
**VISUAL-2**					
20	Right inferior occipital gyrus	42.4	−70.5	−3.1	10,785
21	Left inferior occipital gyrus	−39.2	−76.3	−2.5	5685
**VISUAL-3**					
22	Bilateral lingual gyrus	2.4	−88.8	−7.8	25,952
**VISUAL-4**					
23	Bilateral cuneus	4.5	−88.3	22.4	12,208
**FRONTAL**					
24	Left medial frontal gyrus	−10.9	26.3	−12.0	337
25	Right medial frontal gyrus	20.3	32.1	−13.5	519
26	Left middle frontal gyrus	−24.9	34.7	−14.1	73
**CEREBELLUM**					
27	Bilateral cerebellar tonsil/Culmen	2.4	−45.5	−28.2	34,938
28	Right culmen	10.5	−36.1	−16.1	160
**FRONTOPARIETAL (L)**					
29	Left angular gyrus	−39.7	−57.7	37.1	17,128
30	Left middle frontal gyrus	−42.3	25.7	22.0	13,348
31	Left middle temporal gyrus	−58.2	−49.8	−9.5	577
32	Left cingulate gyrus	−4	22.8	37.3	152
**FRONTOPARIETAL (R)**					
33	Right supramarginal gyrus	51.8	−51.2	34.4	18,758
34	Right middle frontal gyrus	46.1	23.8	27.6	10,309
35	Right middle temporal gyrus	67	−40.8	−3.7	365
36	Right medial frontal gyrus	6.6	29.9	36.2	264
**HIGHER ORDER NETWORK-1**					
37	Bilateral medial frontal gyrus	−2.2	38.6	34.5	9556
38	Left inferior frontal gyrus	−45.9	24.9	−9.1	266
**HIGHER ORDER NETWORK-2**					
39	Left precuneus	0.4	−56.5	48.1	19,104
40	Right inferior parietal lobule	60.3	−34.5	26.0	4480
41	Left inferior parietal lobule	−57.8	−37.2	27.1	3312
42	Left middle frontal gyrus	−30.2	35.8	29.5	1464
43	Right middle temporal gyrus	58.2	−58.4	1.4	542
44	Right middle frontal gyrus	32.8	42.4	25.0	448
45	Left inferior temporal gyrus	−53.2	−66	−0.4	163
46	Left middle occipital gyrus	−38.2	−82.7	20.8	88
47	Left cingulate gyrus	−10.8	−32.5	31.9	88
48	Right middle temporal gyrus	48.9	−72.7	14.4	72
**HIGHER ORDER NETWORK-3**					
49	Right precuneus	32.6	−70.2	33.3	8336
50	Left superior occipital gyrus	−29.8	−78.9	26.3	2736
51	Right posterior cingulate	15.4	−55.9	7.7	2048
52	Right middle frontal gyrus	28.8	8.3	47.5	368
53	Left precuneus	−9.2	−72.6	39.3	208
54	Left lingual gyrus	−10.7	−58	5.1	120
55	Right culmen	25.3	−38.3	−16.1	80
56	Left posterior cingulate	−15.9	−62.5	11.3	72

Three separate classifiers were built that each focused on a separate functional connectivity-based feature.

### Representativeness of RSN (REP)

The dual regression method (Zuo et al., 2010) was used, with the thresholded RSN map as a template, to extract participant-level component maps. The participant-level component maps were then standardized into Z-score maps. As a measure of RSN representativeness, the average Z-score was calculated for each node region in the 16 RSNs, for each participant. This resulted in 56 REP features.

### Between-RSN connectivity (B-RSN)

To obtain a measure of functional connectivity between networks, each group-ICA map was regressed against each participant's 4D dataset to extract the time-course corresponding to that component. Functional connectivity was computed as the temporal correlation between each pair of RSN time-courses. We employed this procedure, as opposed to calculating the correlation between every pair of nodes within any two RSNs and using the average correlation, to extract the time-course corresponding to the network as a whole. In this way, we are able to avoid extracting correlations that may arise from components in a node's time-course that do not correspond to its parent network. This procedure resulted in 120 B-RSN features.

### Within-RSN connectivity (W-RSN)

To compute functional connectivity within an RSN, reference time courses from each of the node regions within a network were generated by averaging the time courses of all voxels within the region. Subsequently, correlation coefficients were computed between each pair of node time-courses within each RSN. As we wanted to analyze node pair connectivity merely in the context of a given RSN, only pairs of nodes *within the same network* were analyzed. Two RSNs contained only a single node each (Visual-1 and Visual-3), and were therefore excluded from this classifier. This resulted in 119 correlation W-RSN features.

### Support vector machine (SVM) classifier

**Figure 2:**
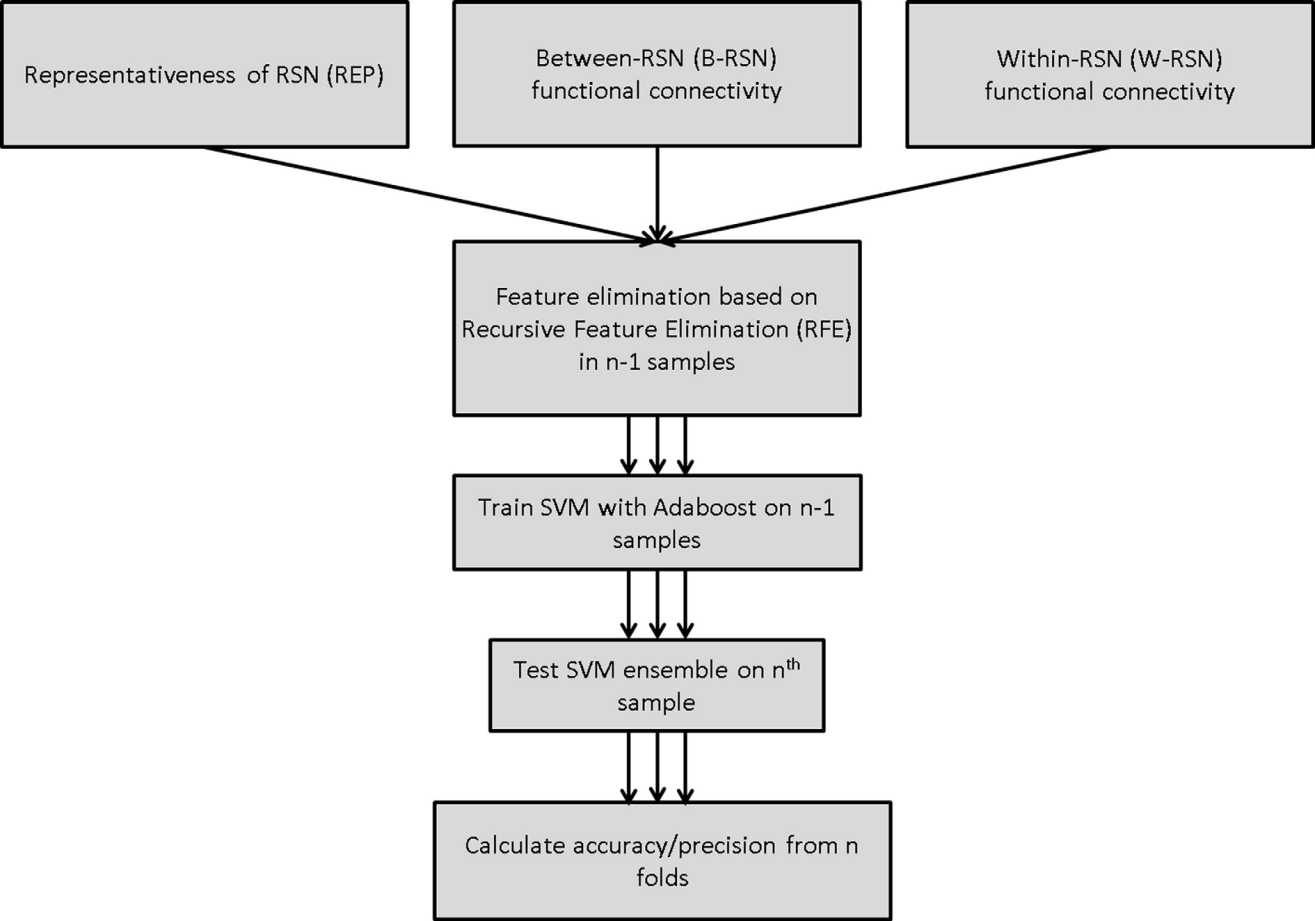
**Classification algorithm for predicting smoking status using SVM-Adaboost**.

SVM training and testing were carried out using the Scikit-learn package in Python (Pedregosa et al., 2011), which is an implementation of the LIBSVM package (Chang and Lin, 2011). A linear SVM was employed in all models (with soft margin parameter *C* = 1). Classification performance was tested using leave-one-out cross-validation (LOOCV). On each run, training data was first scaled, and the corresponding scaling transformation was repeated on the test dataset. Feature selection was carried out prior to classifier-training through recursive feature elimination (Guyon et al., 2002) with either 0, 50, or 90% feature elimination; this provided a comparison of performance with no, medium, and high degree of feature elimination. Features deemed critical by this method were carried forward to the classifier-training stage (Figure 2). Without feature elimination, even with superior classification performance, it would be impossible to make any meaningful inferences about the underlying neurobiology owing to the large number of features involved. Narrowing the set of features to 10% of the original set permits a more detailed understanding of the key circuits involved.

### Adaboost

The linear SVM classifier was supplemented by a boosting algorithm—AdaBoost (Freund and Schapire, 1995). This algorithm involves an iterative process of training the SVM classifier on a *weighted* set of samples, where the weights are determined by the accuracy of the classifier for those samples on the previous iteration. The final classification is obtained through a linear combination of individual classifiers, where the classification of each SVM classifier is weighted by its performance accuracy. In this manner, AdaBoost builds a non-linear classifier ensemble from a weighted combination of multiple linear SVM classifiers.

### Classification performance

To ascertain the performance of a classifier, we calculated accuracy and precision, defined as below:

$$\begin{array}{l} \text{Accuracy}=(number\;of\;true\;positives\\ \;+number\;of\;true\;negatives)/(number\;of\;all\;samples)\\ \text{Precision}=(number\;of\;true\;positives)/\\ \;(number\;of\;true\;positive+number\;of\;true\;negatives) \end{array}$$

To test whether classification performance was significantly above chance, we randomly classified each participant as a smoker or non-smoker, and trained and tested each classifier on this dataset. This process was executed 1000 times to obtain random distributions of accuracy and precision. *Z*-tests were then performed between the actual accuracy/precision values and the generated random distributions to determine statistical significance. Additionally, we tested whether actual accuracy and precision scores were 2 standard deviations above the mean of the generated random distribution.

To identify features maximally contributing to improved classification performance with the within-RSN classifier, we extracted features that were utilized in the classifier following 90% feature elimination on 15 or more runs of LOOCV. Each feature had a chance of 1/119 of appearing in the critical 12 on each run. Features that showed up in 15 or more runs of LOOCV were therefore appearing far more frequently than would be predicted by chance (*p* < 0.000007).

## Results

Smokers and controls did not differ statistically in age, gender, or ethnicity (Table 1), reducing the probability that the classifier's performance was biased by demographic features irrelevant to nicotine addiction. After applying the SVM-AdaBoost algorithm to 21 smokers and 21 controls, accuracy and precision were calculated for the REP, between-RSN, and within-RSN classifiers with and without feature elimination.

Based on the above metrics, we concluded that the within-RSN and REP classifiers can reliably be used to classify smokers from non-smokers (Table 3). On the other hand, the between-RSN classifier's performance was not consistently above chance. This suggests that there is limited predictive information for nicotine addiction in the functional connectivity between RSNs, at least based on the current method of defining network nodes.

**Table 3:**
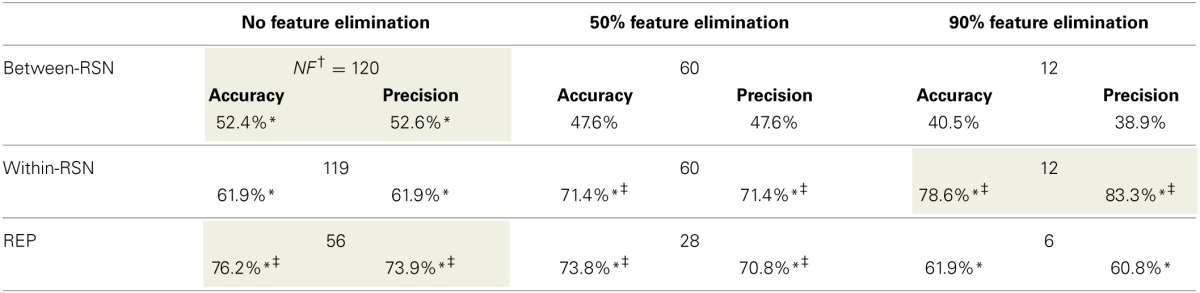
**Performance (accuracy and precision) of the three SVM-AdaBoost Classifiers**.

Best performance for a classifier is highlighted in gray.

^†^NF, number of features.

^*^indicates classification performance was significantly above chance (p < 0.0001).

^‡^indicates classification performance was over 2 standard deviations above chance.

**Figure 3:**
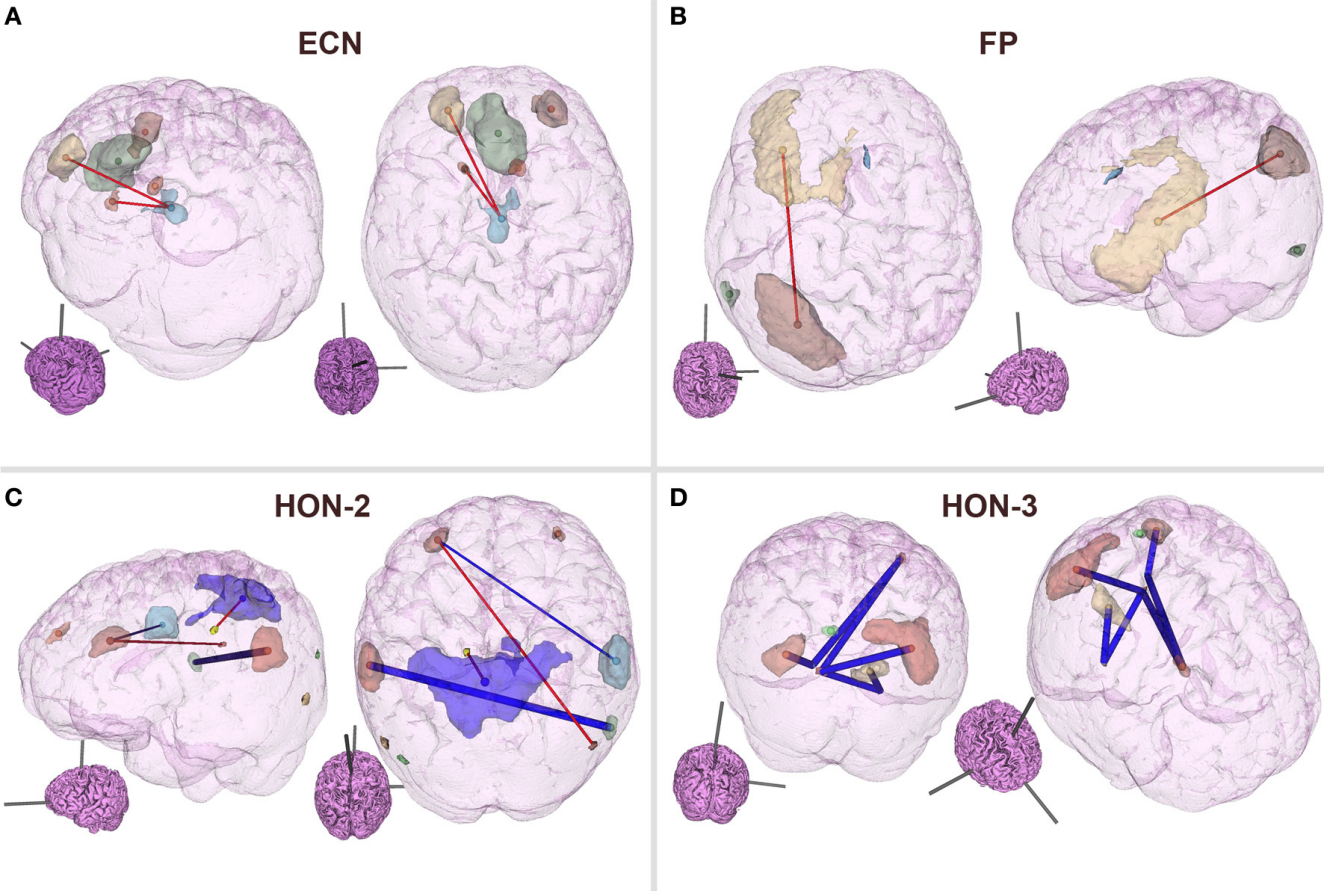
**Features maximally contributing to SVM classification performance**. Features that were utilized in the within-RSN classifier following 90% feature elimination on 15 or more runs of LOOCV were identified, and these consisted of circuits within the **(A)** ECN, **(B)** FP, **(C)** HON-2, and **(D)** HON-3. Red and blue lines indicate circuits in which connectivity was greater and lower, respectively, in smokers relative to controls. Thick lines indicate circuits that were individually statistically different between smokers and controls, as inferred from *t*-tests. Inset brains indicate the orientation of the larger configuration (ECN, Executive Control Network; FP, Frontoparietal Network; HON, Higher Order Network).

As can be observed from Table 3, classification performance significantly improved with feature elimination for the within-RSN classifier. To identify the features maximally contributing to this improved classification performance, we extracted the features that were utilized in the classifier following 90% feature elimination on 15 or more runs of LOOCV (Figure 3). This process revealed that connectivity within HON-3 (6 circuits), HON-2 (4 circuits), executive control (2 circuits), and frontoparietal (1 circuit) networks specifically were predictive of smoking status. These circuits involved parts of the middle and superior frontal gyri, posterior cingulate cortex, precuneus, middle temporal gyri, and inferior parietal gyri.

To confirm that our results were not artifacts induced by head motion, we computed the RMS displacement change for smokers and controls and verified that the two groups did not differ statistically on this measure for translational [*t*_(40)_ = −0.58; *p* = 0.564] or rotational [*t*_(40)_ = 0.86; *p* = 0.394] head motion. Further, the two groups did not differ statistically in the final number of volumes that were included in the computation of correlation co-efficients [*t*_(40)_ = −1.97; *p* = 0.056. Mean_smokers_ = 173.67 ± 9.67; Mean_non-smokers_ = 178.19 ± 3.43].

## Discussion

We employed a machine learning-based approach to identify functional connectivity measures that are predictive of nicotine dependence. A comparison of three network-centered functional connectivity measures revealed that the functional connectivity between nodes within resting state networks is most informative in predicting nicotine dependence. Classification based on functional connectivity *between* these networks, on the other hand, resulted in performance accuracy not consistently above chance levels. It should be noted, however, that classification performance of the within-network classifier improved when many features were eliminated; this suggests that *specific* within-network circuits, or a combination thereof, warrant further investigation in the context of nicotine addiction. We are not implying that within-network connectivity in general would be more informative about the disease.

The REP assessments were comparably successful in predicting smoking status vis-à-vis the within-RSN classifier. However, a similar examination of the critical nodes is difficult as classification performance did not improve with feature elimination, suggesting that all 56 nodes (Figure 1; Table 2) need to be considered when predicting smoking status. As the REP measure indicates the extent to which a specific node behaves like the network in general, strong classification performance here might reflect differences in within-network connectivity in smokers.

On further examination of the within-network connectivity classifier, we found that HON RSNs, including the frontoparietal and ECNs, were critical to the classification process. Within these networks, functional connectivity of the middle/inferior frontal gyrus, posterior cingulate cortex, and precuneus were especially informative regarding nicotine dependence, i.e., were utilized in the classification processes following 90% feature elimination on 15 or more runs of LOOCV. That predictive features were observed in frontal regions is not surprising. Multiple lines of research suggest impairments in this area as being critical to addiction (Goldstein and Volkow, 2002; Volkow et al., 2002; Hester and Garavan, 2004; Kober et al., 2010). The differences observed here may reflect impairments in frontal disinhibition, a deficit thought to be critical to the disease (Goldstein and Volkow, 2011).

Network-centered approaches have identified several large scale networks whose functional connectivity patterns at rest strongly correspond with network activity during various cognitive processes (Smith et al., 2009). Of these, two sets of networks have been implicated in task performance—task-positive networks which are engaged during task performance, such as the ECN, and the task-negative “default mode network” (DMN). The DMN decreases in activity during task performance relative to its baseline at rest (Greicius et al., 2003). These task-positive and task-negative networks exhibit an anti-correlated relationship (Fox et al., 2005). Importantly, the degree to which activity between these networks is anti-correlated predicts variability in performance on cognitive tasks (Kelly et al., 2008), suggesting that the integrity of this between-network coordination is critical to efficient cognitive function. Our data suggests a breakdown of normal connectivity patterns within the ECN and DMN RSNs. This difference in functional connectivity could have two important implications: (1) it could influence the manner in which each network responds to a cognitive task or pharmacological manipulation (e.g., Cole et al., 2010), and consequently the observed interaction between the two networks; (2) similarly the within-network connectivity differences could affect ECN-DMN homeostasis under high-craving states. In support of such a possibility, DMN-ECN connectivity is weakened during smoking abstinence (Lerman et al., 2014). In other words, although the differences observed here are within a network, under various manipulations, they could manifest as between-network effects. (It is tempting to infer that the PCC-related circuits that showed up frequently as critical features speak to a DMN involvement along exactly these lines. However, in our hands, the DMN RSN itself does not appear to be a key player in the classification process, and the PCC-related circuits observed here were located in other higher order RSNs.) Here, the data were from smokers who were allowed to smoke *ad libitum* and were thus unlikely to be experiencing withdrawal. To address whether the connectivity disruptions reflect frontal disinhibition problems or DMN-ECN antagonism differences, classification performance needs to be compared between abstinent and sated smokers. Frontal disinhibition problems are seen to predate addiction (De Wit, 2009; Ersche et al., 2010, 2012), and are exacerbated by chronic drug use (Volkow et al., 2009), but are unlikely to be affected by acute effects of nicotine (Bekker et al., 2005). Thus, middle/inferior frontal circuits should still provide important information for classification in both sated and abstinent states. On the other hand, DMN-ECN dynamics should be markedly different in the two states, and thus functional connectivity within the ECN in one state should not be particularly informative to the other.

It is important to note that some of the features critical to the classification performance may be changes induced by chronic nicotine consumption, while others are likely pre-existent. Parts of the middle frontal gyrus, for example, have been shown to decrease in gray matter volume as a function of lifetime exposure to cigarette smoke (Brody et al., 2004; and is suggested in Gallinat et al., 2006). Importantly, classification performance in this study was guided by a *combination* of different neural features, and not driven by any one feature in particular. In support of this assertion, within-network classification dropped in performance when the number of features was limited to 1 (accuracy = 57.14%, precision = 56.0%). Nicotine addiction is likely an interaction of pre-existing vulnerabilities and nicotine-induced impairments; while machine learning allows us to uncover such interactions, future work will need to disentangle individual contributions from both these sources.

Classification performance here was not as high as has sometimes been reported from rsFC data (see Dosenbach et al., 2010, for example, in which the authors achieved classification accuracy over 90% when classifying individuals as either children or adults); there may be a couple of reasons for this. Firstly, great care was taken here to eliminate head motion-induced artifacts that have previously been shown to influence functional connectivity estimates (Power et al., 2012). Efforts included removal of time points where head motion was above a stringent threshold, inclusion of head motion parameters as nuisance regressors, and a comparison of head motion data between the two groups. By diminishing head motion-related confounds, we have likely reduced any artificial enhancement of classification performance from irrelevant motion artifacts. Secondly, we followed an agnostic approach of including networks/nodes that are involved in a wide range of cognitive processing, and not merely those shown to be distinguishing features within the same sample set. In this way, we have avoided any inadvertent enhancement of classification performance through “double dipping” (Kriegeskorte et al., 2009). Finally, the sample sizes used here, although standard for machine-learning approaches to fMRI data (Zhang et al., 2005; Yang et al., 2010), may have been less sufficient for probing neural differences more subtle than those seen in schizophrenia or Parkinson's disease.

To obtain a more complete picture of disruptions in functional connectivity as a consequence of nicotine addiction, it would be illuminating to examine such data on a continuum of addiction severity. Although we analyzed the data here as binary classes of “high severity of nicotine addiction” and “no nicotine addiction,” additional insight could be gleaned by using support vector regression, for example, to extract features predictive of the FTND (Fagerström et al., 2012). Such a regression-based approach would allow for a more nuanced understanding of individual differences in the severity of addiction, especially when partnered with different treatment strategies. However, as this is a relatively crude measure of disease severity, it is likely that a much larger sample size and variance in FTND would be necessary for such a regression approach. Similarly, support vector regression with a focus on lifetime smoking exposure could provide valuable insights into the consequences of chronic nicotine consumption.

One potential concern regarding our findings is that, currently, there is limited data supporting the value of within-RSN centered analysis in nicotine addiction research. As already mentioned, a previous publication from our lab (Sutherland et al., 2012) approached the consequences of nicotine-related changes in within- (and between-) DMN and ECN connectivity. Prior to this, Cole et al. (2010) examined the effects of nicotine replacement therapy within- (and between-) DMN and ECN connectivity. However, although studies like these that focus on RSNs as defined in Smith et al. (2009) are not common, it is not unusual for addiction studies to focus on within-network connectivity in pre-specified networks. For example, many rsFC studies in addiction have limited their analyses to frontal and mesocorticolimbic circuits (see Sutherland et al., 2012, for a review).

Machine learning based approaches offer both basic and clinical applications for addiction research: the capability to identify neural features critical to predicting addiction, and the potential for using such a classifier in clinical settings to predict treatment outcome or future substance dependence. For the latter, classification performance need approach 95–100% accuracy (Orrù et al., 2012). Although, for screening future substance dependence, high sensitivity (the true positive rate) is likely more important, even at the expense of specificity (true negative rate), than overall accuracy. In any case, a potential limitation to this study is that rsFC by itself may not be powerful enough to predict smoking status with close to perfect performance. Perhaps by including task-based data, or features involving other modalities—e.g., genetics—we may obtain superior predictive capabilities. It has been shown that by combining fMRI and genetics information, classification of schizophrenics from controls can be significantly enhanced (Yang et al., 2010). Similarly, by including genetic information that has previously been shown to differentiate smokers from non-smokers (Kreek et al., 2005; Hong et al., 2010), perhaps the predictive power of such classifiers can be augmented to the extent required for clinical applications. Nevertheless, our data suggests that there is tremendous potential in combining rsFC data with machine learning-based techniques for advancing our understanding of network-level predictive differences critical to addiction.

### Conflict of interest statement

The authors declare that the research was conducted in the absence of any commercial or financial relationships that could be construed as a potential conflict of interest.
